# Supplementary figures and images for: Multiplexed ISSR genotyping by sequencing distinguishes two precious coral species (Anthozoa: Octocorallia: Coralliidae) that share a mitochondrial haplotype
Source: PeerJ. 2019 Oct 4;7:e7769. doi: 10.7717/peerj.7769 (PMC6779117; doi:10.7717/peerj.7769)

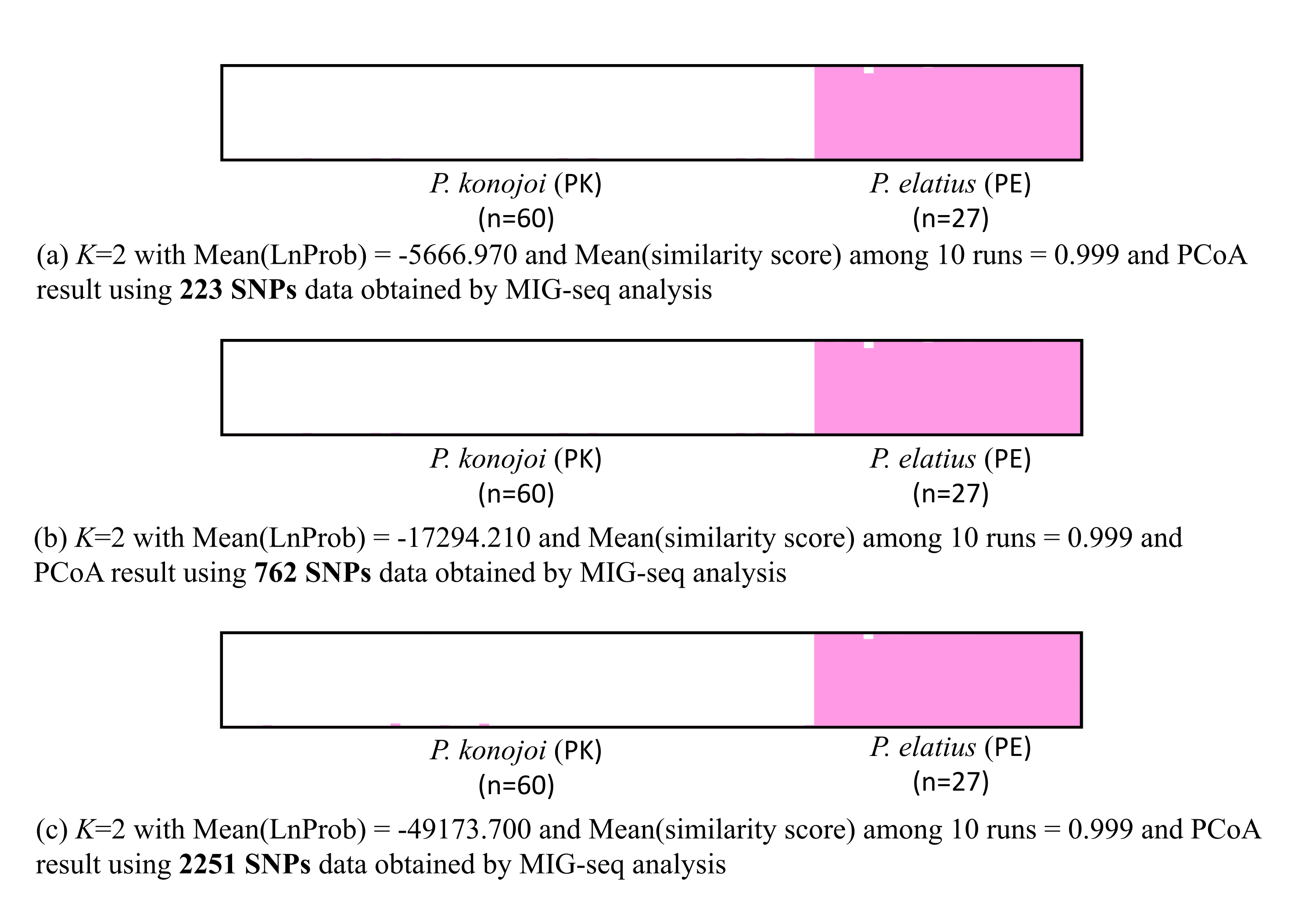

Supplement: Supplemental Information 5 [file peerj-07-7769-s005.png]
